# Early N-terminal pro-B-type natriuretic peptide is associated with cardiac complications and function during pregnancy in congenital heart disease

**DOI:** 10.1007/s12471-021-01540-3

**Published:** 2021-02-03

**Authors:** A. S. Siegmund, P. G. Pieper, B. J. Bouma, F. M. Rosenberg, H. Groen, C. M. Bilardo, D. J. van Veldhuisen, M. G. Dickinson

**Affiliations:** 1 Department of Cardiology, University Medical Centre Groningen, University of Groningen, Groningen, The Netherlands; 2 Department of Cardiology, Heart Centre, Amsterdam UMC, Amsterdam Cardiovascular Sciences, University of Amsterdam, Amsterdam, The Netherlands; 3 Department of Epidemiology, University Medical Centre Groningen, University of Groningen, Groningen, The Netherlands; 4 Department of Obstetrics, University Medical Centre Groningen, University of Groningen, Groningen, The Netherlands

**Keywords:** Congenital heart disease, N‑terminal pro-B-type natriuretic peptide, Pregnancy, Cardiovascular complications, Right ventricular function

## Abstract

**Background:**

Elevated *N*-terminal pro-B-type natriuretic peptide (NT-proBNP) levels at 20 weeks’ gestation predict adverse cardiovascular (CV) complications during pregnancy in women with congenital heart disease (CHD). To improve early risk assessment in these women, we investigated the predictive value of first-trimester NT-proBNP for CV complications and its association with ventricular function during pregnancy.

**Methods:**

Pregnant women with CHD, previously enrolled in a prospective national study or evaluated by an identical protocol, were included. Clinical data, echocardiographic evaluation and NT-proBNP measurements were obtained at 12, 20 and 32 weeks’ gestation. Elevated NT-proBNP was defined as > 235 pg/ml (95th percentile reference value of healthy pregnant women in the literature).

**Results:**

We examined 126 females (mean age 29 years). Elevated NT-proBNP at 12 weeks was associated with CV complications (*n* = 7, 5.6%, odds ratio 10.9, *p* = 0.004). Arrhythmias were the most common complication (71%). The negative predictive value of low NT-proBNP to exclude CV complications was 97.2%. In women with CV complications, NT-proBNP levels remained high throughout pregnancy, while a decrease was seen in women without CV complications (*p* < 0.001 for interaction between group and time). At 12 weeks, higher NT-proBNP levels were associated with impaired subpulmonary ventricular function (*p* < 0.001) and also with a decline in subpulmonary ventricular function later in pregnancy (*p* = 0.012).

**Conclusions:**

In this study, first-trimester NT-proBNP levels were associated with adverse CV complications and a decline in subpulmonary ventricular function later in pregnancy in women with CHD. Early NT-proBNP evaluation is useful for tailored care in pregnant women with CHD.

## What’s new?

- Elevated *N*-terminal pro-B-type natriuretic peptide (NT-proBNP) levels in the first trimester are associated with adverse cardiovascular complications and a decline in subpulmonary ventricular function later in pregnancy in women with congenital heart disease (CHD).
- NT-proBNP levels remain high throughout pregnancy in women with CHD developing cardiovascular complications.
- NT-proBNP levels decrease steadily from the second trimester onwards in women with CHD who do not develop cardiovascular complications.
- First-trimester NT-proBNP evaluation is useful for tailored care in pregnant women with CHD.

## Introduction

In women with congenital heart disease (CHD), several prediction models are available to estimate the risk of maternal cardiovascular (CV) complications during pregnancy [1–3]. However, early prediction of these risks remains challenging. We have previously shown that elevated *N*-terminal pro-B-type natriuretic peptide (NT-proBNP) levels at 20 weeks’ gestation are an independent risk predictor for CV complications in pregnant women with CHD [4]. No data are available about the prognostic value of NT-proBNP in early pregnancy or the (normal) course of NT-proBNP during pregnancy in women with CHD. NT-proBNP is primarily secreted when abnormal ventricular wall stress and volume overload occur, both of which can occur in early pregnancy [5, 6]. From a pathophysiological point of view, early NT-proBNP detection makes sense in CHD patients. Many women with (corrected) CHD start pregnancy with impaired cardiac function, and it is likely that haemodynamic changes during pregnancy expose them to the risk of cardiac maladaptation from the first trimester. Moreover, pregnancy can be associated with persisting structural cardiac remodelling, recurring arrhythmias and deterioration in ventricular function (in particular right/subpulmonary ventricular function) [7–10]. Subpulmonary ventricular dysfunction is an independent predictor for CV complications in pregnant women with CHD [4] and is known to be correlated with elevated NT-proBNP in non-pregnant patients [11, 12]. Identification of impaired cardiac function as early as possible might be effective in preventing CV complications later in pregnancy.

To improve early risk assessment in pregnant women with CHD, we aimed to investigate the predictive value of first-trimester NT-proBNP for CV complications later in pregnancy and its association with ventricular function during pregnancy.

## Methods

### Study design

This present study comprised pregnant women enrolled in the prospective multicentre observational cohort ZAHARA III study (*Zwangerschap bij Aangeboren HARtAfwijkingen*, pregnancy in congenital heart disease) or evaluated by an identical protocol (during standard patient care in our university medical centre). The study design of the ZAHARA III study has been reported previously [13]. Pregnant women with CHD, aged ≥ 18 years, presenting in one of the participating centres at ≤ 14 weeks’ gestation and with NT-proBNP measurements available (≤ 14 weeks’ gestation) were eligible for enrolment in the current study. All the participating centres received the approval of the medical ethics committee and all women (prospectively enrolled) provided written informed consent.

### Pregnancy follow-up and CV complications

At the first ante-partum visit, preconception baseline characteristics of pregnant women were recorded, including: underlying heart disease, CV and obstetric history, age, cardiac status (including New York Heart Association functional class, modified World Health Organisation risk class for maternal risk of CV complications [3], electrocardiogram, laboratory results and echocardiographic recordings), medication use and intoxications. Evaluation of pregnancy was performed at 12, 20 and 32 weeks, including clinical evaluation, standardised echocardiogram, electrocardiogram and laboratory evaluation. All echocardiograms were performed according to an identical ZAHARA study protocol [14], and chamber quantification, valvular function, and systolic and diastolic ventricular function were assessed according to the current guidelines [15–17]. Systemic ventricular dysfunction was defined as ejection fraction < 45% and subpulmonary ventricular dysfunction was defined as tricuspid annular plane systolic excursion (TAPSE) < 17 mm. Consistency and accuracy of the echocardiography data were checked by A.S.S. NT-proBNP at 12 weeks was considered abnormal if the value exceeded the 95th percentile reference values according to gestational age in healthy pregnant women (> 235 ng/ml) [18].

CV complications were evaluated during pregnancy and up to 6 months post-partum. Primary CV complications were defined as the need for an urgent invasive CV procedure, heart failure (according to the guidelines and documented by the attending physician [19]), new-onset or symptomatic tachy- or bradyarrhythmia requiring new or extended treatment, thromboembolic events, myocardial infarction, cardiac arrest, cardiac death, endocarditis and aortic dissection.

### Statistical analysis

Data are presented as mean ± standard deviation, median (25th to 75th percentiles) or numbers (percentages). For the comparison of dichotomous variables, we used the χ^2^ test or Fisher exact test, as appropriate. Univariable logistic regression was used to assess associations between predefined preconception cardiac function parameters, elevated NT-proBNP at 12 weeks and primary CV complications.

NT-proBNP levels during pregnancy were log-transformed to create a normal distribution for further statistical analyses. Uni- and multivariable linear regression models were used for associations between NT-proBNP and cardiac function parameters based on the literature [20]. Variables associated with the studied end points (*p* < 0.10) were entered in the multivariable model. The final model was constructed by backward deletion. Women with a systemic right ventricle were excluded from analyses with subpulmonary ventricular function.

For longitudinal analyses (at 12, 20 and 32 weeks), analyses using generalised estimating equations (GEE) were performed with an unstructured correlation matrix, and data are presented as mean with standard error. For longitudinal comparisons within groups (women with vs without CV complications), an interaction (time × group) was included in the model. Statistical analyses were performed using SPSS version 23.0 (SPSS, Chicago, IL, USA).

## Results

### Baseline characteristics

In the ZAHARAIII study, 204 pregnant women were included initially. Eleven women were excluded, because of a miscarriage (*n* = 6), absence of CHD (*n* = 4) or withdrawal of informed consent (*n* = 1). In the current study, only pregnant women with NT-proBNP ≤ 14 weeks available were included, resulting in a study population of 126 pregnant women. Underlying CHD and baseline characteristics are presented in Tab. 1. Maternal age at conception was 28.8 ± 4.2 years, body mass index was 23.8 ± 3.9 kg/m^2^, and 62 (49.2%) women were nulliparous. Prior CV events were reported in 19 (15.1%) women. Eighteen women had a history of arrhythmia and one woman had a history of transient ischaemic attack.

**Table 1:** Demographics and clinical data

	*n* = 126
Primary CHD	
Left-sided lesions	41 (32.5)
– Repaired aortic coarctation	18 (14.3)
– Aortic valve stenosis/bicuspid aortic valve	18 (14.3)
– Mitral valve disease	5 (4.0)
Right-sided lesions	34 (27.0)
– Double-chambered right ventricle	1 (0.8)
– Ebstein’s anomaly	5 (4.0)
– Pulmonary atresia	1 (0.8)
– Pulmonary valve stenosis	14 (11.1)
– Tetralogy of Fallot	13 (10.3)
Shunt lesions	36 (28.6)
– Abnormal pulmonary venous return	1 (0.8)
– Atrial septal defect	10 (7.9)
– Atrioventricular septal defect	7 (5.6)
– Ventricular septal defect	18 (14.3)
Connective tissue disorder	3 (2.4)
Right aortic arch	1 (0.8)
Complex CHD	11 (8.7)
– Fontan circulation	1 (0.8)
– Transposition of great arteries (Mustard/Senning correction)	3 (2.4)
– Transposition of great arteries (arterial switch correction)	3 (2.4)
– Congenitally corrected transposition of great arteries	2 (1.6)
– Right ventricular hypoplasia with pulmonary valve stenosis and bilateral Glenn procedure	1 (0.8)
– Truncus arteriosus	1 (0.8)
NYHA class	
– I	111 (88.1)
– II	9 (7.1)
Modified WHO class^a^	
– I	16 (12.7)
– II	87 (69.0)
– III	23 (18.3)
Mechanical valve prosthesis	7 (5.6)
Prior cardiac event (HF, TIA, stroke, arrhythmia)	19 (15.1)
Hypertension before pregnancy	5 (6.1)
Pacemaker	2 (1.6)
Cardiac medication use prior to pregnancy^a^	28 (30.1)
– Beta-blocker	21 (22.6)
– Other	13 (14.0)
Echocardiographic parameters^a,b^	
Systemic AV valve regurgitation^c^	3 (3.7)
Pulmonary AV valve regurgitation^c^	6 (7.3)
Pulmonary stenosis^d^	5 (6.1)
Pulmonary valve regurgitation^c^	12 (14.6)
Aortic valve stenosis^d^	7 (8.5)
Aortic valve regurgitation^c^	2 (2.4)
Systemic ventricular systolic dysfunction^e^	3 (3.7)
Systemic ventricular hypertrophy^f^	6 (7.3)
Subpulmonary ventricular systolic dysfunction^g^	17 (20.7)

*AV* atrioventricular, *CHD* congenital heart disease, *HF* heart failure, *NYHA* New York Heart Association, *TAPSE* tricuspid annular plane systolic excursion, *TIA* transient ischaemic attack, *WHO* World Health Organisation

Data are reported as *n* (%)

^a^≤ 1 year before pregnancy

^b^Available in 82 (65.1%) women

^c^Moderate or severe regurgitation

^d^Peak gradient ≥ 36 mm Hg

^e^Ejection fraction < 45%

^f^Systemic ventricular mass/body surface area > 95 g/m^2^

^g^TAPSE < 17 mm

### CV complications and first-trimester NT-proBNP

CV complications were observed in 7 (5.6%) women. Five (71.4%) women had arrhythmias (mainly supraventricular tachyarrhythmias), for which cardioversion and/or increased metoprolol dosage was required. Underlying heart diseases were: mitral valve disease, aortic valve stenosis, transposition of great arteries (Mustard/Senning correction) and Fontan circulation. Arrhythmias occurred between 12 and 28 weeks of pregnancy, except for one woman who had arrhythmia 3 months post-partum. Two (28.6%) women had thromboembolic complications for which anticoagulation therapy was required; one woman (with Mustard correction) had deep vein thrombosis at 23 weeks; and one woman (with connective tissue disorder) had arterial thrombosis at 38 weeks. NT-proBNP was measured at 11.5 (10–13) weeks. Women with CV complications had higher NT-proBNP at 12 weeks compared with women without complications, 301 (117–381) ng/ml versus 95 (53–170) ng/ml (*p* = 0.008). Four women (57.1%) with CV complications had NT-proBNP > 235 ng/ml. Of all women, 86.5% had NT-proBNP ≤ 235 ng/ml (89.1% of women without CV complications). NT-proBNP ≤ 235 ng/ml at 12 weeks had a negative predictive value of 97.2% for the occurrence of CV complications after 12 weeks and a positive predictive value of 23.5%. The sensitivity of NT-proBNP > 235 ng/ml was 57.1% and the specificity was 89.1%. In Tab. 2, univariable regression analyses for the identification of predictors for CV complications during pregnancy are presented.

**Table 2:** Logistic regression analysis of preconception values, elevated *N*-terminal pro-B-type natriuretic peptide (*NT-proBNP*) in first trimester and cardiovascular complications during pregnancy

Variables	OR	95% CI	*p*-value
*Univariable predictor*			
Maternal age at conception	0.9	0.8–1.1	0.401
WHO class III or IV	14.0	2.5–77.9	*0.003*
Parity	0.4	0.1–2.0	0.243
Smoking^a^	2.7	0.3–27.6	0.407
Resting heart rate^a^	1.0	0.9–1.1	0.979
Sinus rhythm^a^	0.4	0.1–1.7	0.202
Use of cardiac medication^a^	3.2	0.7–15.4	0.140
TabelPrior cardiac event	4.8	1.0–23.6	0.052
History of arrhythmia	5.2	1.1–25.5	*0.042*
Mechanical valve prosthesis	9.1	1.4–59.1	*0.020*
Right systemic ventricle	15.5	2.1–114.4	*0.007*
Aortic valve stenosis^a^	5.6	0.9–36.5	0.072
Pulmonary valve stenosis^a^	2.9	0.3–30.4	0.371
Aortic valve regurgitation^a^	−18.8	0–0	0.999
Pulmonary valve regurgitation^a^	−19.0	0–0	0.999
LVEF^a^	1.0	0.9–1.2	0.780
TAPSE^a^	0.8	0.7–1.0	0.100
Subpulmonary ventricular dysfunction (TAPSE < 17 mm)^a^	4.9	0.6–37.7	0.124
Elevated NT-proBNP in first trimester^b^	10.9	2.2–54.1	*0.004*

*CI* confidence interval, *LVEF* systemic ventricular ejection fraction, *NYHA* New York Heart Association, *OR* odds ratio, *TAPSE* tricuspid annular plane systolic excursion, *WHO* World Health Organisation

^a^ ≤ 1 year before pregnancy

^b^NT-proBNP > 235 pg/ml

**Fig. 1:**
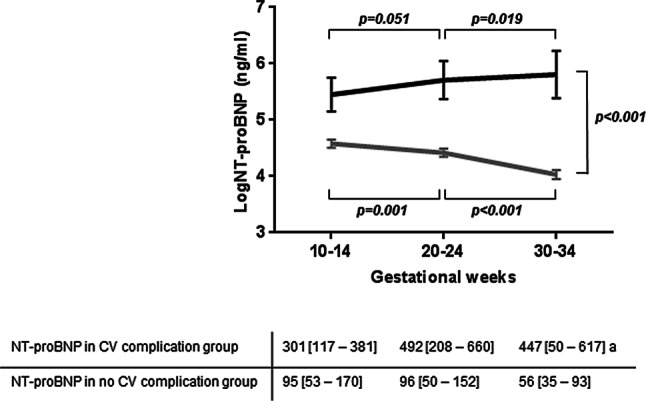
Serial analyses of log-transformed *N*-terminal pro-B-type natriuretic peptide (*logNT-proBNP*) levels throughout pregnancy in women with cardiovascular (*CV*) complications (*black line*, *n* = 7) and without CV complications (*grey line*, *n* = 119). Absolute NT-proBNP levels at 10–14, 20–24 and 30–34 weeks are reported as median (Q1–Q3). ^a^Reported as median (minimum-maximum). A significant difference in the course of logNT-proBNP was found between women with CV complications and without CV complications (*p* < 0.001). NT-proBNP levels decreased after 10–14 weeks’ gestation in women without CV complications and remained high in women with CV complications

Fig. 1 presents the course of NT-proBNP throughout pregnancy of women with and without CV complications based on GEE analyses of log-transformed values, corrected for correlation within subjects. An overall difference in the course of NT-proBNP was found between women with and without CV complications (*p* < 0.001 for interaction between group and time). More specifically, in women without CV complications NT-proBNP decreased during pregnancy but not in women with CV complications. In women with CV complications, NT-proBNP increased between 20–24 and 30–34 weeks (*p* = 0.019).

### Cardiac function and first-trimester NT-proBNP

**Fig. 2:**
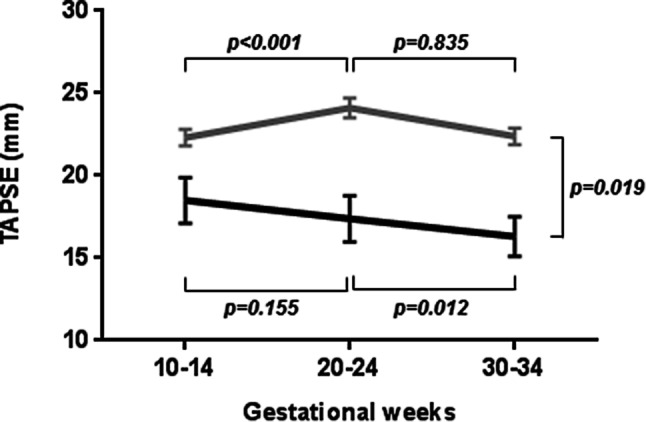
Patterns of tricuspid annular plane systolic excursion (*TAPSE*) during pregnancy in women with elevated first-trimester *N*-terminal pro-B-type natriuretic peptide (NT-proBNP) levels (*black line*, *n* = 12) and women with normal first-trimester NT-proBNP levels (*grey line*, *n* = 92). A significant difference in the pattern of TAPSE was found between women with elevated NT-proBNP and those with normal NT-proBNP (*p* = 0.019). TAPSE increased from 10–14 weeks to 20–24 weeks in women with normal NT-proBNP. Only in women with elevated NT-proBNP did TAPSE significantly decrease from 20–24 weeks’ to 30–34 weeks’ gestation

All correlations between cardiac function and NT-proBNP at 12 weeks are presented in Tab. 3. With multivariable analyses, higher maternal age and reduced subpulmonary systolic ventricular function (TAPSE) remained associated with higher NT-proBNP (*p* = 0.004 and *p* < 0.001, respectively). Fig. 2 presents the patterns of TAPSE of women with elevated and normal NT-proBNP at 12 weeks. A difference in the pattern of TAPSE was found (*p* = 0.019 for interaction between group and time). In women with normal NT-proBNP, TAPSE increased between 10–14 weeks’ and 20–24 weeks’ gestation (22.3 ± 0.5 to 24.1 ± 0.6, *p* < 0.001) but not in women with elevated NT-proBNP (18.5 ± 1.4 to 17.4 ± 1.4, *p* = 0.155). Only in women with elevated NT-proBNP did TAPSE decrease from 20–24 weeks’ to 30–34 weeks’ gestation (17.4 ± 1.4 to 16.3 ± 1.2, *p* = 0.012).

**Table 3:** Associations between log-transformed N-terminal pro-B-type natriuretic peptide, age and cardiac function at first trimester

	*n*	Beta	95% CI	*p*-value
Age	126	0.044	0.013–0.075	* 0.005*
NYHA class	126	0.379	0.041–0.718	* 0.028*
LVEDD/BSA	99	0.044	−0.008–0.096	0.100
LVESD/BSA	92	0.033	−0.016–0.083	0.187
LAVi	50	0.023	−0.010–0.055	0.163
LVEF	103	−0.037	−0.059–−0.016	* 0.001*
TAPSE^a^	104	−0.054	−0.080–−0.029	*<0.001*
S’ RV^a^	74	−0.059	−0.113–−0.005	* 0.034*
E/A ratio	86	0.286	−0.069–0.641	0.113
E’	75	0.021	−0.042–0.084	0.515
*Multivariable regression analyses*^a,b^				
Age	126	0.054	0.018–0.089	* 0.004*
TAPSE	104	−0.060	−0.087–−0.033	*<0.001*

*BSA* body surface area, *CI* confidence interval, *E ’*early diastolic tissue Doppler velocity of systemic ventricular annular ring, *E/A ratio* early to atrial mitral inflow velocity ratio, *LAVi* indexed systemic atrial volume, *LVEDD* systemic ventricular end-diastolic diameter, *LVEF* systemic ventricular ejection fraction, *LVESV* systemic ventricular end-diastolic diameter, *NYHA* New York Heart Association, *S’* systolic tissue Doppler velocity of subpulmonary ventricular annular ring, *TAPSE* tricuspid annular plane systolic excursion

^a^Women with systemic right ventricle excluded from analyses

^b^Degrees of freedom = 72

At 12 weeks, 5 (4.0%) women had systemic ventricular dysfunction and 17 (14.0%) women had subpulmonary ventricular dysfunction.

## Discussion

This study focuses on the predictive role of first-trimester NT-proBNP for CV complications later in pregnancy in women with CHD. The main findings are that elevated first-trimester NT-proBNP is associated with adverse CV complications and with a decline in subpulmonary ventricular function during pregnancy. Furthermore, in women with CV complications NT-proBNP remains high throughout pregnancy. This finding is in contrast to that in women without CV complications, in whom NT-proBNP decreases steadily from the second trimester onwards.

Screening for CV complications early in pregnancy makes sense from a pathophysiological point of view, since plasma volume and cardiac output increase, and 75% of these increases occur in the first trimester [21]. We show for the first time that early NT-proBNP evaluation can be useful in risk estimation in pregnant women with CHD. Low first-trimester NT-proBNP seems to indicate that these women have a good chance of adapting to haemodynamic changes during pregnancy and to complete their pregnancy without CV complications. When first-trimester NT-proBNP is elevated, our findings suggest that these women are at higher risk for CV complications in pregnancy. In this study, no heart failure complications occurred, and it is therefore important to underline that elevated NT-proBNP was associated mainly with the occurrence of arrhythmias. A possible explanation could be that heart failure diagnosis was underreported and/or managed temporarily with extra diuretics before fulminant heart failure symptoms could occur. This could be the result of the stricter guideline-driven peri-pregnancy management we strive for in our centres [3]. The association between NT-proBNP and arrhythmias might be explained by the occurrence of volume overload and wall stress during pregnancy, both leading to increased secretion of NT-proBNP and resulting in a trigger for arrhythmias [22]. By identifying women at risk as early as possible in pregnancy, appropriate follow-up visits, possible treatment of arrhythmias or even prevention of such complications could be managed early on and could lead to a decrease in morbidity. In this study, elevated first-trimester NT-proBNP was also present in women without CV complications and was associated with impaired subpulmonary ventricular function. The finding that higher NT-proBNP is associated with worse subpulmonary ventricular function is in line with previous studies conducted in non-pregnant patients with right heart disease [11, 12]. The current results suggest that the subpulmonary ventricle is less well suited to cope with the increased overload (volume and pressure) in addition to the physiological changes during pregnancy. This hypothesis is supported by the fact that we also found a decline in subpulmonary ventricular function later in pregnancy in women with elevated first-trimester NT-proBNP, whereas subpulmonary ventricular function remained stable in women with normal NT-ProBNP. Reduced subpulmonary ventricular function before and during pregnancy is associated with impaired uteroplacental circulation [13, 23, 24], which, in turn, is associated with adverse maternal and neonatal outcome [25, 26]. Therefore, close surveillance of subpulmonary ventricular function in women with elevated first-trimester NT-proBNP may be warranted. Further studies are needed to determine whether the decline in subpulmonary ventricular function predicted by high NT-proBNP is only confined to pregnancy or persists thereafter.

Although the main focus of this study was on first-trimester NT-proBNP, we also investigated the course of NT-proBNP throughout pregnancy. A difference in the course of NT-proBNP was found between women with and without CV complications. In line with the course of NT-proBNP in healthy pregnant women, NT-proBNP decreased in the second half of pregnancy in women without CV complications [18]. This decrease may reflect physiological cardiac adaptation to pregnancy. Conversely, NT-proBNP remained high in women who developed CV complications later in pregnancy. These data might be of additional value for the interpretation of trends in NT-proBNP during pregnancy in CHD women and may suggest that women with persisting elevated NT-proBNP might be at higher risk for CV complications due to cardiac maladaptation to pregnancy. Complication rates in our study were lower than those reported in previous studies [1, 4, 9, 27], which can possibly be explained by improved pre-pregnancy counselling and management of pregnancy of CHD women over the years [3].

### Limitations

The number of CV complications in this study was relatively small, including mainly arrhythmias and no heart failure (worsening). These results are based on a specific heterogeneous study population and individual diseases may be underrepresented. NT-proBNP levels differ per diagnosis and therefore these results might not be representative for each specific type of CHD [20]. Also, negative and positive predictive value are strongly dependent on the prevalence (and type) of CV complications and should be kept in mind when interpreting these results. Lastly, due to our population size and there being no controls available, we used a cut-off point for elevated first-trimester NT-proBNP based on previous data from healthy pregnant women [18]. Larger studies are warranted to identify a cut-off point based on the occurrence of CV complications in pregnant CHD women.

## Conclusion

In the current study, first-trimester NT-proBNP levels were associated with CV complications, particularly arrhythmias, and a decline in subpulmonary ventricular function later in pregnancy in women with CHD. Early NT-proBNP evaluation is useful for tailored care in pregnant women with CHD. NT-proBNP levels remain high throughout pregnancy in women that develop CV complications. In contrast, in women who do not develop CV complications, NT-proBNP levels decrease steadily from the second trimester onwards. Further studies are needed on the predictive value of NT-proBNP for subpulmonary ventricular deterioration during pregnancy in women with CHD.
